# Receding Water Line and Interspecific Competition Determines Plant Community Composition and Diversity in Wetlands in Beijing

**DOI:** 10.1371/journal.pone.0124156

**Published:** 2015-04-07

**Authors:** Zhengjun Wang, Huili Gong, Jing Zhang

**Affiliations:** 1 College of Life Sciences, Capital Normal University, Beijing, China; 2 College of Resources Environment and Tourism, Capital Normal University, Beijing, China; Shandong University, CHINA

## Abstract

Climate and human-induced wetland degradation has accelerated in recent years, not only resulting in reduced ecosystem services but also greatly affecting the composition and diversity of wetland plant communities. To date, the knowledge of the differences in community parameters and their successional trends in degraded wetlands remains scarce. Here based on remote sensing images, geographic information system technology, and statistical methods, we produced a successional gradient map of the Yeyahu Wetland Nature Reserve in Beijing, which has experienced a steady decline in water level in recent decades. In addition, we analyzed community composition and diversity along with each identified gradient. The results showed that community diversity decreases while dominance increases with the progress of succession, with the highest diversity occurring during the early stage of succession. Moreover, the community demonstrates greater similarity among subareas during later successional stages, and the similarity coefficients calculated from the important value (IV) of each species are more accurate. Correlation analysis showed that the impact of soil factors on diversity was not significant at a subarea scale, although these nutrients showed an increasing trend with the community succession. Furthermore, the IVs of the dominant species had a particularly significant impact on diversity, showing a significantly negative correlation with diversity indices and a significantly positive correlation with dominance indices. Further analysis showed that the retreat of water level resulted from sustained drought and local human activities was a major extrinsic driving force resulting in observed differences in the community successional stages, which resulted in differences in community composition and diversity. On the other hand, interspecific competition was the main intrinsic mechanism, which significantly influenced the IVs of the dominant species and community diversity. The results of this study could aid in improving the understanding of community composition, diversity, and its successional trends in degraded wetlands.

## Introduction

Community succession is a central issue in ecological research [1, 2]. Succession is essentially a complex ecological process involving different types of interactions at multiple temporal and spatial scales, including those between plants and their environment and intra- and interspecific interactions and competition within a community. This is particularly observed in the case of long-term community succession, where interactions and ecological processes tend to be more diverse and complex. Elucidating the mechanisms underlying community succession can help understand and predict successional trends, and maintain succession toward an ecosystem state that sustainably meets human needs [2, 3].

Currently, research into community succession has focused on two main approaches, including experimental research and natural community succession investigation and analysis [2]. The purpose of the two approaches is to assess the successional mechanisms and trends of a focused community by analyzing community composition and diversity during different successional stages. However, the first approach implies strong human intervention or control over the experimental design. For example, under the same or similar environmental conditions, the specific plant functional groups were removed from a given community to observe the resultant change in diversity or biomass in one study [4], whereas in another study, new communities with variable species composition were designed and rebuilt to compare the effects of diversity to ecosystem functioning and community succession [5]. In addition, some studies analyzed and assessed community succession mechanisms and trends using mathematical modeling or by controlling environmental conditions such as design of environmental gradients [6, 7]. The latter approach excludes any human impact and focuses exclusively on the succession of the natural state. Several studies have recently focused on natural community succession [8–10]. Typical natural succession, such as that observed during natural ecological restoration of areas within nature reserves, where often grazing exclusion is enforced in grasslands, is a relatively long and complex ecological process. Research into processes of natural succession requires long-term monitoring programs, which are often time-consuming and difficult to implement [11, 12]. For convenience, several researchers have adopted a method considering spatial variability, instead of time [13, 14]. However, a formalized approach for determining successional stages in these studies is lacking, increasing the level of uncertainty in the results.

In community succession, the abovementioned two approaches are necessary for studying the processes and mechanisms of community succession; however, they also have shortcomings. The succession achieved by the experimental design is generally over a shorter duration and can relatively rapidly provide some results and conclusions, whereas the conclusions obtained ultimately need to be verified by natural community succession. Natural community succession is evidently more complex and lengthy, and the conclusions obtained from studying natural succession are more realistic, particularly in case of succession caused by abnormal environmental conditions and human disturbances. However, the impacts caused by several processes and the conclusions obtained during the study of natural succession also need further confirmation by designing targeted experiments. Therefore, using a combination of these two approaches allows us an improved comprehension of community succession in the natural world.

Several studies of succession considering plant species composition and diversity have mainly focused on forest and grassland communities [10,15–18], more specifically on changes in species composition and diversity during ecological restoration [8, 19–21]. In contrast, studies on plant composition and diversity in wetlands, particularly degraded wetlands, are scarce. In general, plant community composition and species diversity in riverine and lacustrine wetland ecosystems often differ from forest and grassland ecosystems. This is particularly true for arid and semiarid areas majorly because of the terrain, soil moisture, and fluctuations in water levels caused by climate change and human activities [22, 23]. These wetland plant communities are generally characterized by habitat-specific distribution patterns such as zonal distribution along the edge of the water body and a gradient distribution perpendicular to the water body [24].

Several studies have demonstrated that surface water plays an extremely important role limiting plant community composition, structure, and diversity in wetlands [25–27]. However, some natural wetlands have recently been exhibiting a continuous trend of degradation due to global warming and climate anomalies particularly associated with the steady decline in water levels due to persistent droughts and human disturbance [28]. These degraded wetlands are frequently transformed to dry lands because of a drop in the water-line, leading not only to the reduction or depletion of ecosystem services provided by the wetlands but also to a change in the direction of plant succession in wetlands [29]. For example, wetland degradation reduces the aquatic plant and bird diversity, disrupts the original soil–water–atmospheric interactions, alters local climatic conditions, and reduces the value of wetland tourism [30]. Furthermore, wetland degradation promotes the invasion of xeric or mesophyte plant species [31].

For degraded wetlands experiencing a continuous decrease in water level, it would be necessary to understand the differences in species composition and diversity among communities at different successional stages and the trend of succession. In addition, further investigation would be required to determine the key factors driving these differences, and ultimately determining the mechanism behind succession in wetland communities.

In the current study, we analyzed the plant community composition and diversity along a successional gradient in the Yeyahu Wetland Nature Reserve in Yanqing, Beijing. Our objectives were (1) to compare the species composition and diversity among plant communities in different subareas with different successional stages and (2) to evaluate the mechanisms underlying the differences observed and the trends of community successional in degraded wetlands.

## Materials and Methods

### Study site

**Fig 1:**
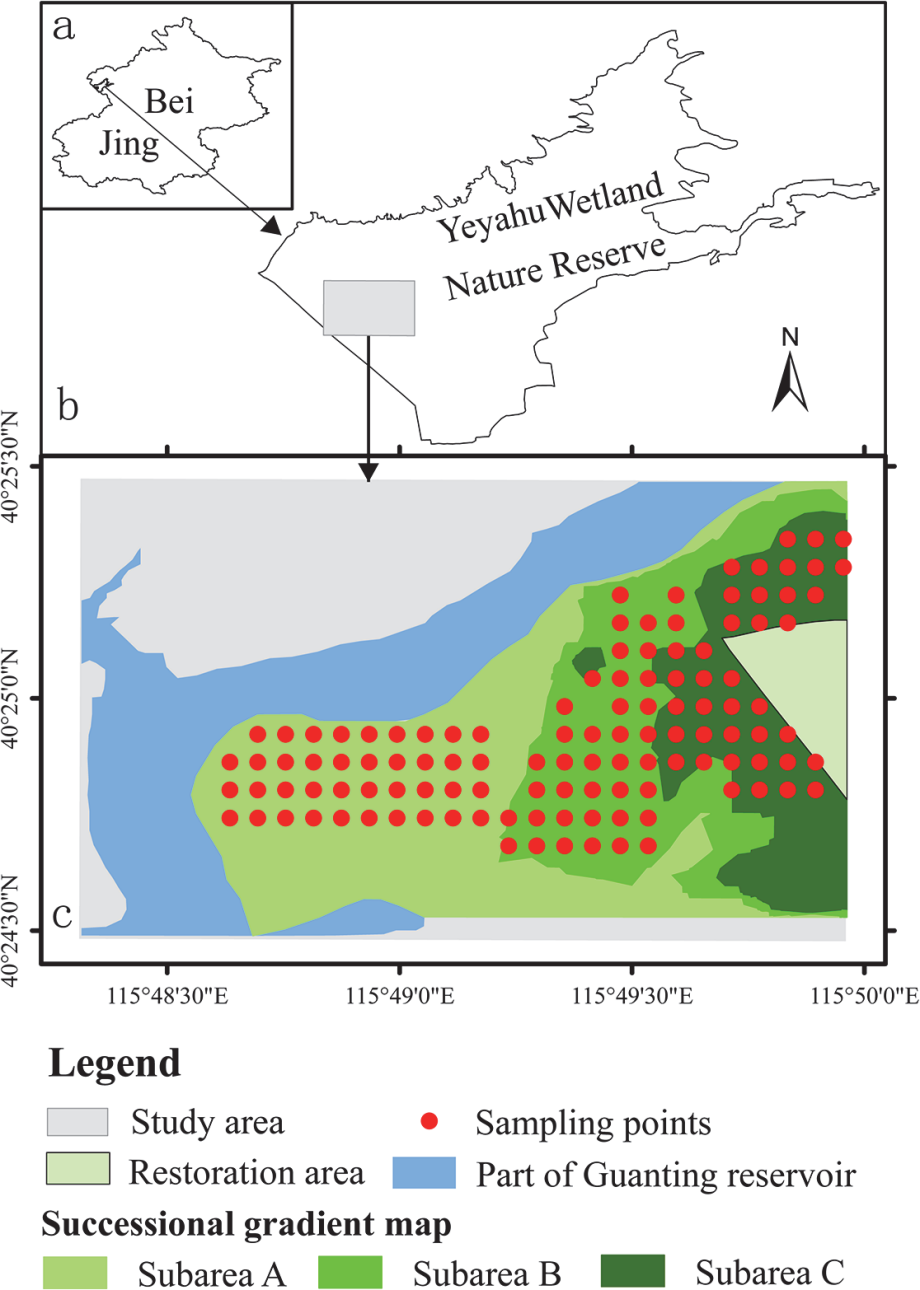
Locations of the study area and the sampling points in the Beijing’s Yeyahu wetland nature reserve. Note: a and b show the location of the study area in the Beijing metropolitan area and the Yeyahu wetland nature reserve, respectively; c shows the successional gradient map and the locations of the sampling points in each subarea.

**Fig 2:**
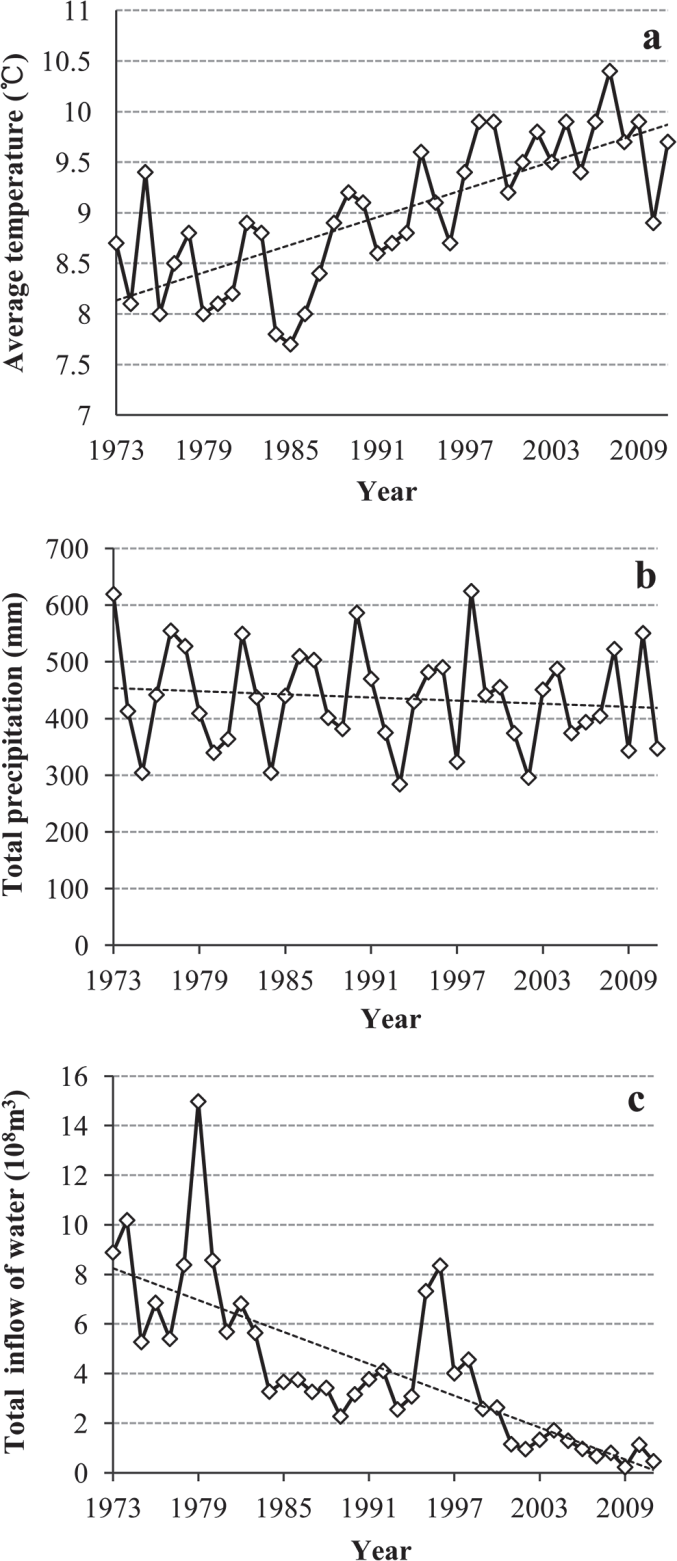
Variations in annual meteorological factors and the amount of water in the Guanting reservoir from 1973 to 2011. Note: a, b and c show annual average temperatures, total rainfall and total inflow of water, respectively.

The study area is located in the Yeyahu Wetland Nature Reserve in the Yanqing County, Beijing (115°48′19″–115°49′58″E, 40°24′29″–40°25′28″N). Surface water within the protected areas originates from the adjacent Guanting reservoir, which was one of the most important water sources for Beijing (Fig 1A and 1B). However, due to the impact of agricultural and industrial effluent, the water quality of the reservoir and Yeyahu wetland has continued to deteriorate since the late 1980s, and the reservoir was withdrawn as a source of drinking water for the city in 1997. In addition, numerous reservoirs have been built upstream, and water extracted by industry and agriculture has increased. The strengthening of human activities and prolonged droughts (Fig 2A and 2B) has resulted in a sharp decrease in inflow of water (Fig 2C), decline in the water level, and formation of the secondary wetlands. Furthermore, the decrease in water levels has resulted in a marked decrease in the quantity of aquatic plants, animals, and birds as well as a decline in the wetland ecosystem services [32].

Although the wetland is in a state of degradation, a highly diverse community and lush vegetation remains, which is a habitat for a rare waterfowl and a transfer station for migratory birds [33–35]. The formerly inundated areas, which are now exposed, present the richest diversity of plant species in the entire protected area, where *Phragmites australis* and *Hemarthria altissima* are the two dominant plant species. *P*. *australis* is mainly distributed to the west of the study area, whereas *H*. *altissima* grows largely to the east of the region.

The regional climate can be classified as continental monsoon, which alternates between very cold and dry during winter to hot and rainy during summer [36]. Yearly records (Fig 2A and 2B) show an average annual temperature of approximately 9°C. The average annual rainfall in the region is approximately 436 mm, with most rainfall occurring from July to September.

### Production of a successional gradient map

To comparatively analyze community composition and species diversity along a successional gradient in a degraded wetland, we were first required to define the boundary for each of the exposed areas presenting different successional stages. Recent developments in remote sensing technology and the availability of remote sensing images provide a reliable means to construct a gradient map showing these boundaries. In this study, we used Landsat Thematic Mapper (TM) and Enhanced Thematic Mapper Plus (ETM+) data, spanning 1973–2011, to define the extension of the areas exposed due to the annual retreat of the water line. Landsat archival images were obtained from the remote sensing ground station, China Academy of Sciences, and the US Geological Surveys Global Visualization Viewer (http://glovis.usgs.gov). These images were preprocessed using a geometrical correction to the WGS 84 coordinate system (some of the corrected images did not require this process). To produce this map, we first obtained a vector map for each year showing the exposed areas, which were identified by visual interpretation of the satellite image. Each vector map was subsequently converted into raster format, with each grid interpreted as a value of 1 or 0 for an exposed or flooded area, respectively. Subsequently, the raster maps were superimposed, providing a cumulative exposure time (here, referred to as “successional time”) for each grid cell. Based on available satellite images, we reclassified the integrated map into three successional gradients, named subareas A, B, and C, with cumulative exposed time of up to 8 years, 13 years, and 20 years, respectively. Considering the dates that these satellite images were obtained and their time span as well as the most recent water levels, the actual successional time of three subareas may have varied in a range of 8–10, 13–31, and 20–39 years for subareas A, B, and C, respectively. This map was then reconverted into the vector format (Fig 1C) for subsequent analyses. The visual interpretation of the satellite images and the production of the successional gradient map were performed with the support of Esri ArcGIS 10.0 software (Esri Inc., 1999–2012).

Species composition and diversity and variables such as plant coverage were compared among subareas as defined by the successional gradient map.

### Sampling design and measurement of soil variables

The area was systematically sampled to assess plant diversity between August and September during 2011, when plant growth remains relatively stable. A total of 117 quadrats were established, separated by 85 m in the east-west directions and 111 m in the north-south direction. A total of 39 sampling quadrats were established per subarea (Fig 1C). To analyze the natural processes underlying community succession, restoration areas were not considered in this study. Each quadrat area was 1 m^2^, and plant species, abundance, coverage, and aboveground biomass (dry weight) for each plant species were recorded per quadrat. The geographic and environmental information including the elevation, coordinates, and thickness of litter for each quadrat were recorded, and certain soil physical and chemical variables including water content, total nitrogen (TN), total phosphorus (TP), available nitrogen, available phosphorus, soil organic matter, and pH were also measured for each sampling location. Soil samples were oven- and air-dried for measurement of water content and chemical variables, respectively. TN was determined by the semimicro Kjeldahl method, and TP was measured using HCLO_4_–H_2_SO_4_ digestion and the molybdenum–antimony–ascorbic acid colorimetric method. Levels of available nitrogen, available phosphorus, and soil organic matter were determined by the Olsen, Alkali-diffusion and Tyurin’s methods, respectively. The soil pH was measured by a pH meter (Professional Meter PP-20, Sartorius Company, Germany). The Yeyahu Wetland Nature Reserve of Yanqing County, Beijing provided permission to conduct the investigation of plant community composition and diversity.

### Diversity indices

Plant diversity was calculated using Margalef’s richness index (represented by R in the following formulas) [37], Shannon–Wiener’s diversity index (H′) [38], Pielou’s evenness index (E) [39–40], and Simpson’s dominance index (D) [39, 41], as expressed below.

Richness index:

R=(S−1)/lnN (1)

Shannon index:

$$H'=-\sum_{i=1}^{S}p_{i}\ln p_{i}$$ (2)

Evenness index:

E=H'/lnS (3)

Simpson index:

$$D=\sum_{i=1}^{S}\frac{n_{i}(n_{i}-1)}{N(N-1)}$$ (4)

In the above equations, *S* denotes the number of species in a community, *N* is the total number of individuals, *n*_i_ the number of individuals from species *i*, and *p*_*i*_ the proportion of the individuals belonging to species *i*.

**Fig 3:**
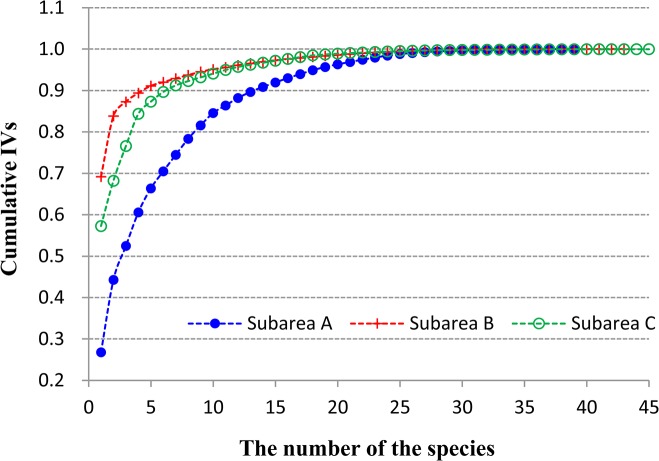
Variation in the cumulative IVs for each community in the three subareas.

The important value (IV) can be regarded as an indicator of the growth condition and dominance of each species within the studied community. For relative coverage, relative density, and relative frequency of each species, the arithmetic means were considered as the IV of the species in the corresponding community (For each quadrat, we used the relative biomass of each species instead of the relative frequency) [42], and the species presenting the highest IVs were considered as the dominant species in the community. Here the relative coverage of each species is expressed as the ratio of the species coverage to the total coverage (relative values of the other variables were also calculated using the same method). To understand the differences in species composition among the three defined subareas, the contributions of each species to the cumulative importance values are shown in Fig 3. The IV also determined the order in which each species was added to the cumulative value; thus, the species whose IV was the highest was added first [43].

### Similarity Analysis

The Jaccard similarity coefficient was used to compare the level of similarity between communities, following the formula [44]:

$$q=\frac{c}{\left(a+b+c\right)}$$ (5)

Here *q* represents the similarity coefficient, *c* is the number of species present in both communities, *a* represents the number of species only present in the community *A*, and *b* represents the number of species only present in the community *B*. The improved Morisita–Horn index by Wolda [45] was used to analyze community similarity based on species abundances as follows:

$$CMH=2\sum \frac{\left(ani\cdot bni\right)}{(da+db)\cdot aN\cdot bN}$$ (6)

$$da=\sum \frac{{\left(ani\right)}^{2}}{aN^{2}}$$ (7)

$$db=\sum \frac{{\left(bni\right)}^{2}}{bN^{2}}$$ (8)

Here *an*_*i*_ and *bn*_*i*_ are the number of individuals from species *i* in community *A* and *B*, respectively (in this study, the IV was used instead), and *aN* and *bN* are the number of species in community *A* and *B*, respectively.

### Analysis of interspecific competition

We analyzed and compared the results of interspecific competition at the subarea and quadrat scales (Tables 1 and 2). At the subarea scale, we used the IVs of each species calculated by the whole subarea level, whereas the IVs of each species within each quadrat were used at the quadrat scale. In this study, the IV was regarded as an indicator and evaluation index used for comparison of the competition results between two species, and the species with a higher IV was regarded as the winner of the competition at the corresponding scale. In addition, we summed the number of quadrats in which each species won the competition in each subarea for comparing the competition results at the subarea scale.

**Table 1:** Comparisons of competitive ability between *Phragmites australis and Hemarthria altissima* at the subarea scale.

Items used for Comparison	IV in subarea level			Number of qudrards in which the highest IVs occurred		
Subareas	A	B	C	A	B	C
*Phragmites australis*	0.16	0.13	0.09	13	3	1
*Hemarthria altissima*	0.13	0.49	0.41	6	33	31
Sum	0.28	0.62	0.5	19	36	32

**Table 2:** Comparisons of competitive ability between *Phragmites australis and Hemarthria altissima* at the in quadrat scale.

The quadrats in which the two species cooccurred	Only in the quadrats in which the IVs of the two species were the first and second highest			All quadrats in which the two species cooccurred		
Subareas	A	B	C	A	B	C
Number of the quadrats in which the two species cooccurred	2	25	18	14	34	31
Number of the quadrats in which the IV of *Phragmites australis* is higher	0	3	1	7	5	2
Number of quadrats in which the IV of *Hemarthria altissima* is higher	2	22	17	7	29	29
Average IV of *Phragmites australis*	0.22±0.09	0.19±0.02	0.15±0.02	0.17±0.02	0.16±0.02	0.11±0.02
Average IV of *Hemarthria altissima*	0.53±0.10	0.67±0.04	0.73±0.04	0.28±0.06	0.66±0.04	0.66±0.04
Sum of the IVs of the two species	0.75±0.01	0.86±0.02	0.88±0.02	0.45±0.06	0.82±0.03	0.77±0.03

### Statistical analyses

**Fig 4:**
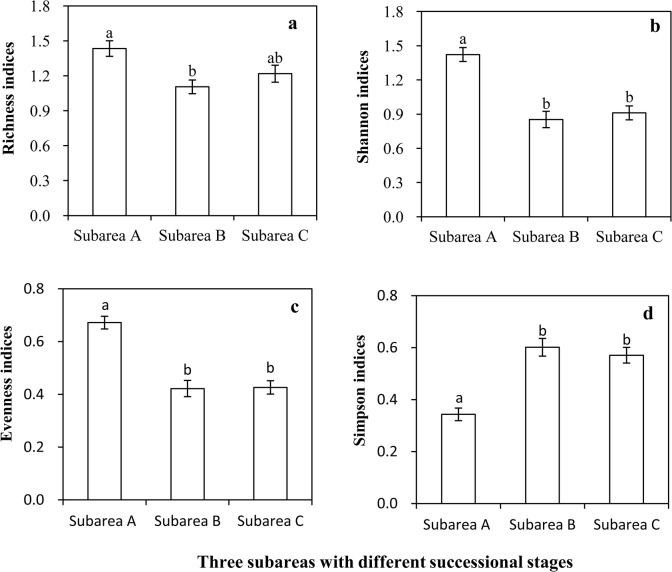
Comparison among diversity indices in the three subareas. Note: a, b, c, and d show richness, Shannon, evenness, and Simpson indices, respectively. Per diversity index, those subareas sharing a common superscript letter were not significantly different; Significance level is P < 0.05.

The data used for diversity analyses were normally distributed and showed homogeneous variance (Simpson indices fulfilled these conditions using a natural logarithm transformation). Thus, the diversity indices among the three subareas and their IVs were compared using a single factor analysis of variance (ANOVA) and post-hoc Tukey test for multiple comparisons (Fig 4 and Table 3). For soil variables, TN, TP, soil water content, and pH were compared and analyzed by the abovementioned methods (TN and soil water content fulfilled normal and homogeneous variance conditions using a natural logarithm transformation). Available nitrogen, phosphorus, and soil organic matter levels were compared using nonparametric test methods owing to their non-normality and heterogeneity of variance (Table 4). The correlation between two variables was conducted using a Pearson correlation analysis with a two-tailed test (Tables 5 and 6). Data analyses were conducted in SPSS 19, and the graphics were drawn in Excel 2007 software.

**Table 3:** Mean value for each parameter measured in each quadrat in the three subareas.

Subarea	Coverage (%)	Above-ground biomass (g/m^2^)	Density (individuals/m^2^)	IV
A	42.4±4.1^a^	185.3±21.0^a^	253.4±26.1^a^	0.50±0.03^a^
B	69.3±3.6^b^	414.0±26.0^b^	434.1±28.4^b^	0.71±0.03^b^
C	77.0±5.0^b^	434.5±24.8^b^	708.3±88.1^c^	0.68±0.03^b^

Note: the values are given as mean ± standard error. IV: Important value of the dominant species. Per column, those values sharing a common superscript letter were not significantly different. Significance level is P < 0.05.

**Table 4:** Comparisons of soil variables and elevations among three subareas.

Parameters	AN	AP	TN	TP	SOM	PH	SWC	Elevation
Subarea A	27.23±1.29^a^	1.19±0.08^a^	0.60±0.03^a^	0.31±0.01^a^	9.35±0.51^a^	8.83±0.04^a^	8.62±0.76^ab^	474.23±0.74^a^
Subarea B	40.46±2.63^b^	2.81±0.48^b^	1.33±0.09^b^	0.33±0.01^a^	16.91±1.35^b^	8.61±0.04^b^	11.03±0.93^a^	472.72±5.25^a^
Subarea C	42.46±2.66^b^	2.12±0.22^b^	1.24±0.07^b^	0.35±0.01^b^	15.32±0.74^b^	8.55±0.04^b^	7.19±0.51^b^	475.62±0.75^a^

Note: the values are given as mean ± standard error. AN: available nitrogen; AP: available phosphorus; TN: total nitrogen; TP: total phosphorus; SOM: soil organic matter; SWC: soil water content. Per column, those values sharing a common superscript letter were not significantly different. Significance level is P < 0.05.

**Table 5:** Correlations between IVs of the dominant species and diversity indices in the three subareas.

Subarea	Richness	Shannon	Evenness	Simpson
A	-0.204	-0.596**	-0.650**	0.647**
B	-0.465**	-0.791**	-0.781**	0.814**
C	-0.304	-0.710**	-0.724**	0.717**

Note: ** Correlation is significant at p < 0.01 (two-tailed)

**Table 6:** Correlations among soil variables, elevation, diversity indices, and the IVs of dominant species in the three subareas.

Subarea	Parameters	AN	AP	TN	TP	SOM	PH	SWC	Elevation
A	richness	-0.20	0.04	-0.08	-0.06	-0.18	-0.27	0.16	-0.17
	shannon	-0.07	0.05	0.05	-0.01	-0.10	-0.20	0.04	-0.17
	evenness	0.00	0.05	0.07	0.09	0.04	-0.15	-0.02	-0.07
	sipmson	0.06	-0.07	-0.06	-0.03	0.02	0.09	0.00	0.09
	YSZ_IV	-0.24	-0.14	-0.07	0.02	-0.05	0.16	-0.04	-0.12
B	richness	0.01	0.19	0.02	0.01	0.01	-0.16	-0.28	-0.13
	shannon	-0.12	0.06	-0.07	-0.03	-0.07	-0.05	-0.28	-0.03
	evenness	-0.13	-0.01	-0.09	-0.02	-0.09	0.01	-0.24	0.01
	sipmson	0.15	-0.04	0.07	-0.02	0.07	0.04	0.28	0.03
	YSZ_IV	0.00	-0.22	-0.16	-0.19	-0.18	0.26	0.03	0.22
C	richness	0.03	-0.10	0.07	0.23	0.09	-0.04	-0.04	-0.22
	shannon	-0.24	-0.14	-0.11	0.20	0.00	0.05	0.03	-0.24
	evenness	-0.28	-0.10	-0.13	0.16	-0.02	0.06	0.02	-0.17
	sipmson	0.22	0.08	0.13	-0.19	0.00	-0.06	-0.03	0.21
	YSZ_IV	0.25	0.17	0.24	-0.08	0.11	-0.20	0.06	0.16

Note: The abbreviations of the parameters see the notes of Table 4.

## Results

### Characteristics of plant communities at different successional stages

The characteristics of the plant communities varied among the three subareas, representing different successional stages. For example, plant coverage, biomass, and density in subarea A were significantly lower than those in subareas B and C. Plant community density in subarea C was significantly higher than that of subarea B; however, the coverage and biomass in these two areas did not differ significantly (Table 3). In addition, the IVs of the dominant species for subarea A were significantly lower than that of the other two subareas (Tukey test, p < 0.05). The IVs for subarea B were higher than that of subarea C; however, this difference was not significantly different (Tukey test, p = 0.767; Table 3).

### Diversity and similarity of plant communities in different successional stages

#### Richness and Shannon indices

Species richness in subarea A was the highest; however, it was not significantly different from that of subarea C. Species richness in subarea B is slightly lower than that of subarea C but this difference was not statistically significant (Fig 4A). The Shannon index average estimated for subarea A was significantly higher than that of subareas B and C; however, the indices of subareas B and C were not significantly different (Fig 4B). This indicates that subarea A, which was in a lower stage of succession compared with other subareas, presented the higher levels of plant diversity, whereas the differences among subareas tended to decrease with the succession of the plant community.

#### Evenness and Dominance

The evenness index in subarea A was significantly higher than that of subareas B and C, but the differences between subareas B and C were not significant (Fig 4C). The Simpson index, which indicates the level of dominance of a species in the community, showed the opposite trend to the evenness index. The level of dominance in subarea A was significantly lower than that of subareas B and C, and the level of dominance in subarea C was slightly lower than that of subarea B, although this difference was not statistically significant (Fig 4D). This indicates that in areas with shorter successional stages, plants were more uniformly distributed and the dominance index was lower; however, the differences among subareas tended to decrease as the succession progressed.

Evenness and dominance in the communities from each subarea can also be confirmed on the basis of direct observations of the contribution of each species to the cumulative IV of all species in the corresponding subarea. As shown in Fig 3, the cumulative IVs in subarea A gradually increased with the increase in species number, whereas those in the other two subareas sharply increased at the beginning, followed by a gradual increase. Moreover, the numbers of species whose IV exceeded 3% in subareas A, B, and C were 10, 4, and 5, respectively, and 17, 28, and 29, respectively, for species below the 1% threshold. This shows that more dominant species appeared in the subareas with the earlier successional stages, and the distribution of their IVs was more even. As the succession progressed, the number of dominant species tended to decrease; some dominant species lost importance in the community and some even disappeared.

#### Community similarity

Species composition varied among subareas A, B, and C, containing 39, 43, and 45 species, respectively. The species similarity coefficient between subareas were 0.64 (subarea A–B), 0.56 (A–C), and 0.60 (B–C). Species composition varied among the three subareas, but the overall differences were not particularly obvious. In contrast, the similarity coefficients, based on the IVs among subareas were 0.58 (subarea A–B), 0.64 (A–C), and 0.96 (B–C). Subareas B and C appeared to be very similar. In addition, the differences in quantitative characteristics among subareas, which provide a more realistic representation of the actual natural communities observed, were more obvious than the differences in species composition. Moreover, plant community compositions and quantitative characteristics tended to become more similar as succession progressed.

### Comparison of soil variables and their relationships with diversity indices

Correlation analysis showed that all considered soil chemical variables were not significantly correlated with diversity indices in three subareas (Table 6). However, these variables have obvious differences among the three subareas. The concentration of TN, soil organic matter, and available phosphorus increased significantly from subarea A to C. The concentration of these variables in subarea A was significantly lower than that of subarea B and C, whereas the latter two showed no significant difference. The maximum of the three variables occurred in subarea B. The variation of available nitrogen and TP were consistent with the abovementioned three variables, but their maximum appeared in subarea C. In addition, PH showed a strong alkaline trend in the entire study area, and alkalinity decreased gradually with succession (Table 4).

### Comparison of interspecific competition among the three subareas

In the three subareas, *P*. *australis* and *H*. *altissima* were the two dominant species in the communities. In subarea A, the IVs of the two species are the largest and second largest, respectively. In subareas B and C, the IVs of *H*. *altissima* were considerably greater than in all other plant species. The IV of *P*. *australis* was the second largest dominant species in subarea B, whereas only slightly lower than the second largest dominant species, *Salsola collina*, in subarea C. Despite the IV of *P*. *australis* being lower at the subarea scale, its IVs at the quadrat scale (mean ± standard error: 0.13 ± 0.02) exceeded that of *S*. *collina* (mean ± standard error: 0.03 ± 0.01) in 18 of 25 quadrats in which the two species occurred simultaneously. This demonstrates that *P*. *australis* are more competitive in the subarea. In addition, the number of quadrats in which the IVs of the two dominant species were the highest, and the size of their IVs showed that their competitive abilities were much higher than all other species (Table 1).

*P*. *australis* and *H*. *altissima* showed different competitive abilities in the three subareas. In subarea A, *P*. *australis* had almost no advantage when the two species appeared in the same quadrat, although *P*. *australis* had a slight overall superiority in the subarea (Tables 1 and 2). In the majority of the quadrats in which the two species occurred simultaneously in subarea B or C, the IVs of *H*. *altissima* were markedly higher than those of *P*. *australis* (Table 2). In addition, from subarea A to C, the IVs of *P*. *australis* gradually decreased, whereas those of *H*. *altissima* significantly increased (Table 1). This demonstrated that the competitiveness of the two species displayed distinct trends along succession. The competitiveness of *H*. *altissima* increased and that of *P*. *australis* decreased.

### The impact of IVs of dominant species on diversity

The average IV of the dominant species was lowest in subarea A (Table 3), indicating that their overall competitiveness was relatively low in this area, allowing for less competitive and rare species to establish and promoting an increase in species diversity. Similarly, the IV in subarea B was larger than that of subarea C, with plant diversity showing its lower values in these two subareas. Furthermore, the IVs were negatively correlated with Shannon, evenness, and species richness indices and these correlations became stronger with the increase of IVs. Shannon and evenness indices showed a significant negative correlation with the IVs, whereas the correlation with species richness was significant only for subarea B (Table 5), indicating that the IVs significantly influence species richness after a certain higher value. In addition, the Simpson indices showed a significant positive correlation with the IVs (Table 5), indicating that as the IVs of the dominant species increase, species dominance also increases and species diversity decreases.

## Discussion

### Determination of subareas and its rationality

We used remote sensing images over 20 years to divide exposed areas into the three subareas A, B, and C, based on cumulative exposed time. Here nearly half of satellite images were not available during the study period. This, however, did not affect our results. The available images accounted for more than half of the studied years and were almost evenly distributed throughout the study period. Although it is difficult to determine the real succession time of communities, the difference of exposure time among the three subareas was determinate, i.e., exposure time of subarea A, B and, C increased along with the succession.

In addition to the limitation of availability of satellite data, the temporal frequency of satellite data measurement is generally inconsistent with that of ground survey data, particularly when considering study periods over decades. Our sampling period was from August to September, and only a portion of the available satellite data was collected during this period of time. We had acquired 13 years of data in the period of time from June to October when the water level was relatively stable; in addition, 7 years of data from other times of the year was obtained. Based on the monitoring data from the Guanting Reservoir, the inflow indicated a tendency of decrease, particularly over the most recent 10 years (Fig 1C). Compared with the interannual variability, the intraannual and interseasonal variations of range for the exposed areas was highly limited if there was no influence of abnormal rainfall, such as heavy rain and floods, in corresponding year. For the satellite data available over 7 years obtained during relatively dry seasons, we carefully examined the dates of image capture and determined that abnormal rainfall did not occur in most years except for 1995. Therefore, most satellite images captured during dry seasons were generally able to reflect the exposed areas, which occurred during the wet seasons in the corresponding year, particularly over the most recent 10 years.

The aim of the study was to assess the impact of retreat of water on plant community succession. Therefore, the partition of subareas was based on the cumulative exposed time and did not consider other variables. According to our observations of remote sensing images and the recent exposed areas, we divided the study area into three subareas. Subarea A was a newer and stable water-subsided area with approximately 10 years cumulative exposed time. Subarea C was the earliest exposed area with the longest cumulative exposed time. The exposed time of subarea B was between that of subarea A and C, with some areas in which the retreat of water and flooding occurred alternatively a few times during the study period. In addition, according to our observation and analysis, community characteristics such as coverage, dominance, and diversity showed obvious differences among the three subareas (Fig 4 and Table 3). The abovementioned analyses demonstrated that our division was reasonable and in line with reality.

### Consideration of sampling size and marginal effect

Whether the data collected by quadrat sampling can be used to analyze community succession depends on whether the size and number of quadrats can sufficiently represent the diversity and characteristics of other community variables in different successional subareas. Quadrat sampling is a conventional method used by many researchers during herbaceous investigation, whereas the number of the quadrat and interval between sampling points may vary across studies [46, 47]. It is difficult to use a uniform standard for sampling, and it requires comprehensive judgment according to the scope of the study area, heterogeneity of the habitat, complexity of the community structure, available time and other possible factors [48, 49]. In recent years, more reliable methods, such as the rarefaction method, have become available to estimate the adequacy of sample size for a given area [50–52]. We used the rarefaction method to verify the adequacy of sample size in the three subareas studied. The result showed that the sample size was sufficiently large for estimating the corresponding diversity index, particularly the Shannon and Simpson indices, in the three subareas (S1 Fig).

Besides sampling size, marginal effects may also influence the results of analysis on community diversity [53, 54]. In the experiment design, we considered and attempted to prevent the marginal effect based on the following aspects: (1) The study area was located in the interior of the protected area, in which human disturbance is strictly controlled, and therefore, the effect of human activity on the plant samples in the marginal parts could basically be ignored. (2) According to our observation, the plant community undergoes a process of slow transformation nearby the boundary between the three subareas, even in the adjacent region between subarea C and the restoration area, where measures of large restoration have not occurred and just excluded human interference. In addition, certain samples collected nearby the lake were at a distance from the water body. Therefore, the influence of the marginal effect on plant diversity should be quite limited in the current study. (3) To further verify the impacts from the marginal effects, we conducted the same analysis as that on the original data by removing five samples nearby the boundary in the three subareas, respectively (S2 Fig). The results showed that the trend and differences of plant diversity among the three subareas were consistent with that of the original data (S1 Table). Moreover, the results from the independent samples t-test also showed that the four diversity indices used showed no significant differences between the original (39 points per subarea) and the new sample points (34 points).

### Effects of environmental conditions on the IVs of dominant species and diversity

From a perspective of long-term community succession, climate change, particularly declining water levels, resulted in the formation of the current subareas undergoing different stages of succession; this is the main reason and external the driving force causing the differences in the community composition, diversity, and IVs of dominant species. However, from the perspective of a short-term and local scale, the soil nutrient content, moisture, litter, and altitude have a direct or indirect influence over the IVs of dominant species.

According to our analysis, the soil variables and diversity indices were not significantly correlated in the three subareas, suggesting that the impact of soil fertility on diversity is mainly manifested by an indirect style in the scale of the study area. That is, soil nutrients indirectly impact diversity by directly influencing plant growth and development, such as the IVs of the dominant species.

The soil contains richer levels of nutrients during the later succession stages, closely related to long-term accumulation of plant litter. Plant litter is an important source of soil organic matter and other nutrients such as nitrogen and phosphorus. According to our preliminary investigation, the average thicknesses of the litter in subarea B and C were 33.3 ± 4.4 cm and 20.11 ± 4.9 cm (mean ± standard error), respectively, with litter being thicker and more widespread in the B area. Subarea A was characterized by almost no litter. A certain thickness of litter is conducive for maintenance of the soil moisture and increasing soil fertility and is thus a significant factor promoting the survival and development of the dominant species which have adapted to the corresponding habitat over long-term succession; however, it greatly hinders certain rare species as well as shorter species that are shaded by taller species and has accordingly curbed the survival of these species and decreased plant diversity. The role of litter on community structure and diversity has been the subject of study reports [55, 56], and our results support the conclusion attained in a study of the grassland community in which the increase of litter cover is often accompanied by a decline in the species richness and evenness [57].

In arid and degraded wetlands, a relatively high soil water content is beneficial for increasing biomass and coverage of species and generally has a more significant role in promoting the increase of dominance, particularly in the areas where a few species occupy the dominant position. For example, soil water content in subarea B was the highest and significantly higher than that of subarea C (p < 0.01). Accordingly, the average IV of dominant species in subarea B was the highest (Table 3). Although soil water content of subarea A was higher than that in the subarea C, there were no significant differences (Table 4). Moreover, the IVs of dominant species were more impacted by the lower coverage than the corresponding soil water content in subarea A where the size of average IV of dominant species has reflected this point (Table 3).

In the degraded wetlands, soil water content was also affected by elevation. Soil water content and altitude showed a significantly negative correlation (p < 0.05). In subarea B, the average elevation was the lowest but the soil water content was the highest. The low altitude not only is beneficial to the accumulation of rain water but also facilitates groundwater intrusion into the topsoil in the relatively low-lying wetlands, thus contributing to maintenance of soil moisture.

### Effects of interspecific competition on the IVs of dominant species and diversity

Interspecific competition is an interaction and inevitably occurs between coexisting species within a community, which compete for a limited environmental resource such as living space, light, water, and soil nutrients by directly or indirectly inhibiting each other [58–59]. If demands on the environmental resource of coexisting species are different, there is niche complementarity, or there are enough resources, then the intensity of competition is weakened and a certain degree of species coexistence is evident, as in the subarea A. If the demands of coexisting species on the environmental resource are equal or similar, then interspecific competition tends to become intense. The result of competition is that the winning party develops and its IV becomes greater, whereas the inferior party is generally curbed or dies out because its living spaces become small and IV decreases, as in the case of subarea B and C.

Interspecific competition can be reflected in the subarea and quadrat scale of the study. At the subarea scale, *P*. *australis* and *H*. *altissima* were in a dominant position in the competition, particularly during the late succession stages, when *H*. *altissima* occupied the shares of the other dominant species and resulted in many species becoming rare during succession (Fig 3 and Tables 1 and 2). At the level of quadrat, under approximation of homogeneous soil conditions, the two main dominant species were the winners of the competition in most cases. When comparing the two species, *H*. *altissima* was generally the winner of the competition. The result of competition was that *H*. *altissima* occupied more space and other species either disappeared or were excluded because their living space become smaller, correspondingly resulting in increased community dominance and decreased diversity.

Differences in competitive ability among species are essentially due to the differences in properties of species and their adaptability to environment. The two dominant species are perennial plants and have well-developed root systems, particularly *P*. *australis*. These features place the two species in a favorable position in the competition. Although both species are hygrophytes, *H*. *altissima* is a more drought-resistant grass and is not suited to the aquatic environment, whereas *P*. *austr*alis can to a certain extent live in a saturated environment. These differences are the reasons for their different distribution patterns. In addition, the areas in a state of later succession stages are nearby newly exposed areas containing greater numbers of *H*. *altissima*, which inevitably exerts a great influence on invasion and settlement of *H*. *altissima* in the newly exposed areas.

In the current study, although we lacked time series data or control experiments for validating interspecific competition, we could still prove the presence of interspecific competition and its changes with community successional stages. First, the successional stage of each subarea was determined. Second, the results of interspecific competition in the various stages were also particularly obvious and changed with succession. In addition, due to influence by periodic retreat of the water level, the initial environment of each exposed area over the different succession stages were similar. For example, numerous aquatic plants disappeared and new exposed areas were more easily invaded and settled by some hygrophyte or mesophyte species at nearby subareas of a later succession stage. The similarity between communities in each subarea has confirmed this. Therefore, each new exposed area experienced almost similar successional process, and the similarity of the process was higher among subareas experiencing a later successional stage. That is, the exposed areas during the early successional stages can be almost considered as the early successional stages in the areas experiencing later successional stages. Some research has in addition showed similar results in that competition tends to occur more in fertile soil, wetland, and stabile habitats, particularly among the herbaceous plant community in the wetlands [60–62].

### Effects of the IVs of dominant species on diversity and its causes

Analysis in this study showed that the IVs of dominant species, compared with other factors, had a particularly significant effect on the composition and diversity of the plant community. As defined above, IV provides a comprehensive measure of species biomass, density, and coverage within a community. The impact of IV on diversity actually reflects the impact of these three variables on diversity. For example, vegetation coverage and density were lower and plant distribution was sparser in subarea A (Table 3), where the successional time was the shortest. In this subarea, plant species receive on average more sunlight and absorb more soil nutrients than plants from other areas, allowing for some relatively small species to grow, promoting plant diversity. Plant coverage and density increased with the community succession. Interspecific competition intensifies in such a situation and some species may gradually be replaced by others by competitive exclusion. Finally, only a few dominant species establish in a competitively dominant position and the overall species diversity decreases. Studies have shown that the earlier established plant species have an inhibitory effect on community dominance during the early succession stages [62]. This also correspondingly results in a higher diversity during early successional stages [1]. Our results are consistent with the abovementioned findings.

### The relationships among the IVs of dominant species, environmental conditions, and interspecific competition

The IVs of dominant species are a reflection of the different adaptability of each species to environmental conditions, and is also the result of interspecific competition within a community. In fact, the IVs of dominant species are jointly determined by interspecific competition and environmental conditions. Environmental conditions are the external impact factor of IVs of dominant species, and interspecific competition is the internal mechanism.

Environmental conditions are filters for the existence of species, but in many cases, the impact of environmental conditions on plant community structure and diversity is realized through interspecific competition, particularly for some plants with the same or similar environmental needs. From a broader perspective of the concept, the adaptability of different species to the environment can also be considered as a reflection of interspecific competition. In addition, the results of the current competition will become the new basis and conditions for a next round of competition and will be more conducive to the survival and development of the dominant species.

### Effects of community succession on similarity

Several studies have only used species composition to compare similarities or differences among different communities [63–65] and have ignored the effects of species quantitative variables, such as coverage, density, or IV on community similarity. Therefore, these results inevitably have some limitations. For example, the community similarity coefficients calculated from the species composition data were similar among communities in the three subareas of the study, but the difference between similar coefficients based on the IVs was very large. This is because subarea B and C contained more rare species, which resulted similarity in species composition and a great difference in quantity variables among each subarea. Compared with the similarity of species, the similarity coefficient calculated by the IVs of species can more accurately reflect the degree of similarity among communities and their actual situation.

According to our analysis, the differences between community similarities among the three subareas mainly resulted from the differences in the stages of succession. Different successional stages resulted in differences in composition, diversity, and similarity among communities. However, with the progress of succession, communities in different subareas tended to be more similar or resulted in homogenization in species composition and their quantitative characteristics.

### Analysis on the reasons for retreat of water

Retreat of water of the Yeyahu wetlands was closely related to inflow of the Guanting Reservoir, which has been affected by climate change and human activities. Based on years of data, the annual average temperature of the Guanting Reservoir showed a trend of increase, whereas the rainfall displayed a downward trend. However, the decrease of inflow into Guanting Reservoir is more obvious than rainfall, particularly over the most recent 10 years (Fig 2B and 2C). According to the investigation, inflow of Guanting is primarily affected by increasing human activities. The rapid economic development in the region has resulted in increased demand for water [32]. For example, several dams and reservoirs have been built in the upstream region, directly affecting the inflow to Guanting Reservoir. This is a main reason for the retreat of water of from the Yeyahu wetlands.

## Conclusion

The present study showed that the three subareas which were considered varied to different degrees in their plant community composition and diversity. The community diversity decreased, whereas the dominance increased with the progress of succession, and the subarea with the highest diversity occurred during the early stages of succession. In addition, the community was more similar among subareas of later successional stages. The similarity coefficients calculated from the IV of each species tended to be more accurate and consistent with actual community variables. Correlation analysis showed that the impact of soil factors on diversity was not significant at the subarea scale, although these nutrients showed an increasing trend with community succession. Moreover, the IVs of the dominant species had a particularly significant impact on diversity. The IVs of dominant species had a significantly negative correlation with diversity indices and significantly positively correlation with dominance indices. Further analysis showed that the retreat of water level resulted from sustained drought, and local human activities are a major extrinsic driving force causing the observed differences in the community successional stages, which resulted in differences in community composition and diversity. On the other hand, interspecific competition was the main intrinsic mechanism significantly influencing the IVs of the dominant species and community diversity.

## Supporting Information

S1 Fig The rarefaction curves for three diversity indices and the variation of diversity indices in the three subareas. Note: a, b, c, d and e show Fisher's alpha diversity (richness index), Shannon, Simpson, variation of alpha diversity and variation of Simpson indices, respectively. In this study, we used the EstimateS 9.1.0 to calculate and analyze corresponding rarefaction curves. The purpose of using the software is only to assess the adequacy of the sample size; therefore we did not consider the consistency of the formulas used for calculating the diversity indices in the study and those that the software provided. According to the S1 Fig, with the increase in the number of samples, Shannon index reached a steady state in the three subareas, and the Simpson index reached the state just in the subarea B and C (S1A, S1B and S1C Fig). In addition, Simpson indices in the subarea A and richness are getting very close to the asymptote according to their variation (S1C, S1D and S1E Fig). Moreover, considering the Simpson and even indices can replace each other to some extent, we did not analyze the changes of evenness index (the software does not involve the index).
(TIF)

S2 Fig Locations of the study area and the sampling points in the Beijing’s Yeyahu wetland nature reserve (for 34 sampling points used for access edge effect). Note: a and b show the location of the study area in the Beijing metropolitan area and the Yeyahu wetland nature reserve, respectively; c shows the successional gradient map and the locations of the sampling points in each subarea.
(TIF)

S1 Table Comparison among diversity indices in the three subareas (for 34 sampling points). Note: Per column, those values sharing a common superscript letter were not significantly different. Significance level is P < 0.05.
(DOC)
