# Supplementary figures and images for: Receding Water Line and Interspecific Competition Determines Plant Community Composition and Diversity in Wetlands in Beijing
Source: PLoS One. 2015 Apr 7;10(4):e0124156. doi: 10.1371/journal.pone.0124156 (PMC4388535; doi:10.1371/journal.pone.0124156)

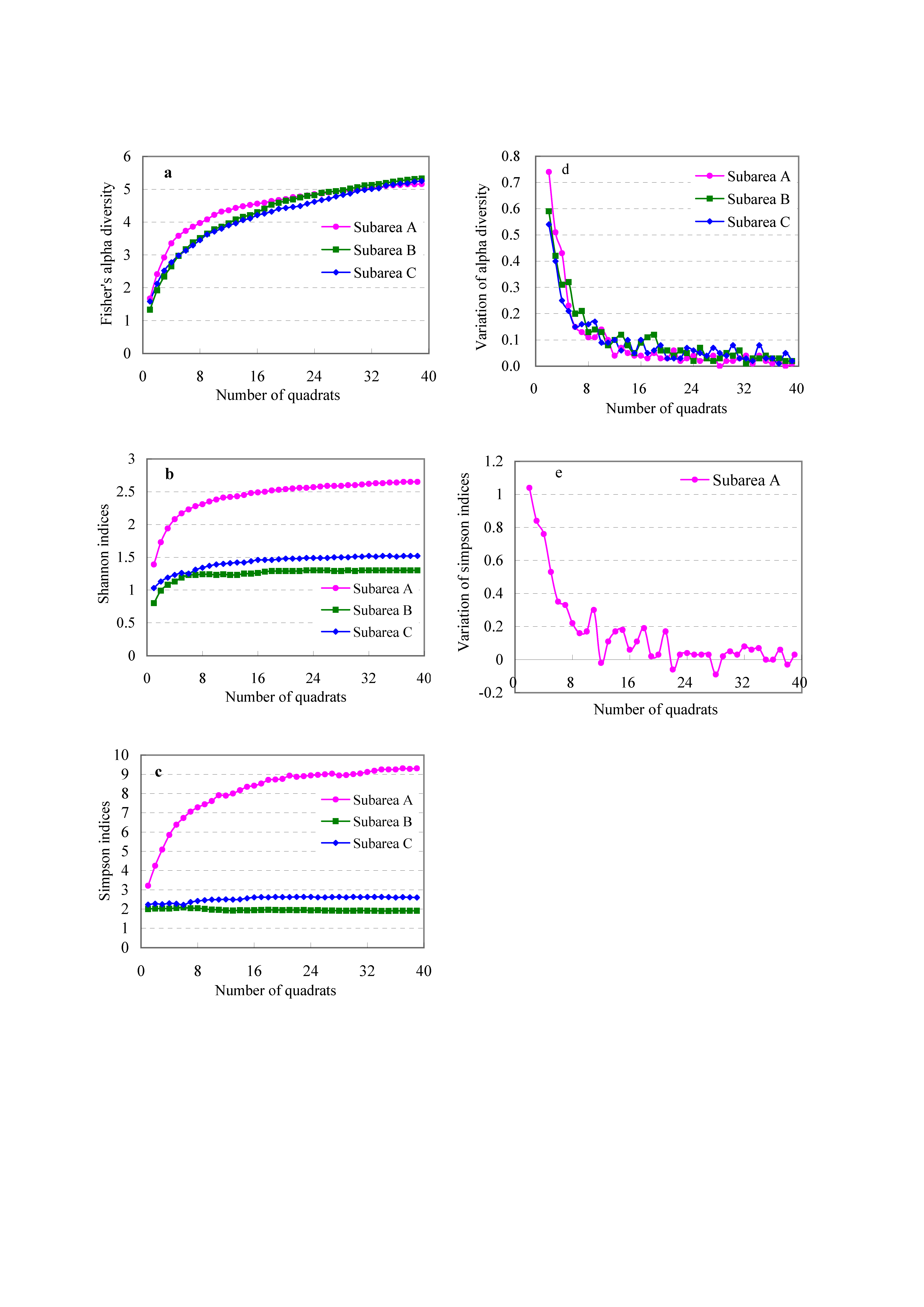

Supplement: S1 Fig — Note: a, b, c, d and e show Fisher's alpha diversity (richness index), Shannon, Simpson, variation of alpha diversity and variation of Simpson indices, respectively. In this study, we used the EstimateS 9.1.0 to calculate and analyze corresponding rarefaction curves. The purpose of using the software is only to assess the adequacy of the sample size; therefore we did not consider the consistency of the formulas used for calculating the diversity indices in the study and those that the software provided. According to the S1 Fig, with the increase in the number of samples, Shannon index reached a steady state in the three subareas, and the Simpson index reached the state just in the subarea B and C (S1A, S1B and S1C Fig). In addition, Simpson indices in the subarea A and richness are getting very close to the asymptote according to their variation (S1C, S1D and S1E Fig). Moreover, considering the Simpson and even indices can replace each other to some extent, we did not analyze the changes of evenness index (the software does not involve the index). (TIF) [file pone.0124156.s001.tif]

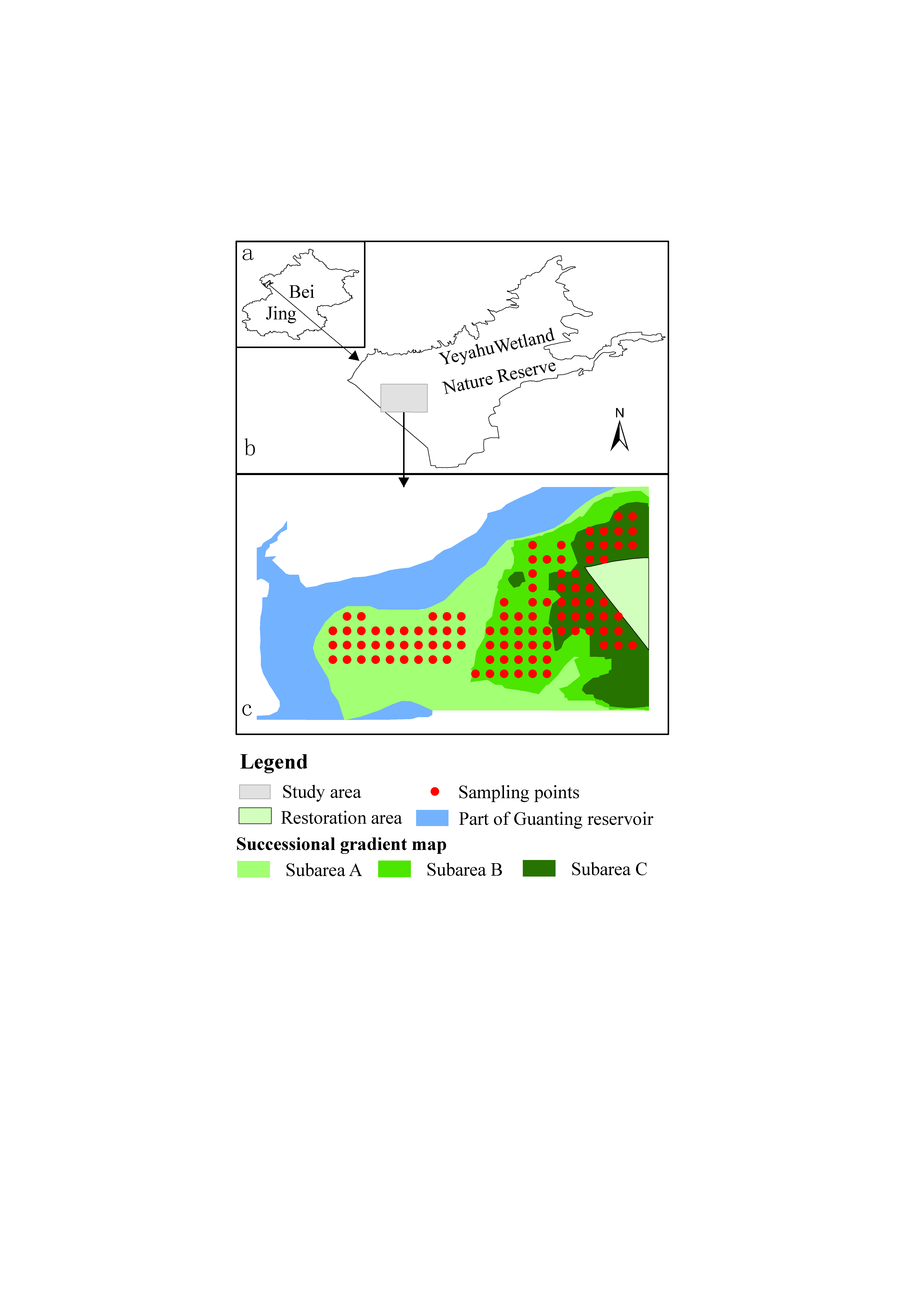

Supplement: S2 Fig — Note: a and b show the location of the study area in the Beijing metropolitan area and the Yeyahu wetland nature reserve, respectively; c shows the successional gradient map and the locations of the sampling points in each subarea. (TIF) [file pone.0124156.s002.tif]
