# Supplementary material for: Receding Water Line and Interspecific Competition Determines Plant Community Composition and Diversity in Wetlands in Beijing
Source: PLoS One. 2015 Apr 7;10(4):e0124156. doi: 10.1371/journal.pone.0124156 (PMC4388535; doi:10.1371/journal.pone.0124156)
Supplement: S1 Table — Note: Per column, those values sharing a common superscript letter were not significantly different. Significance level is P < 0.05. (DOC) [file pone.0124156.s003.doc]

| Subarea | Richness | Simpson | Shannon | Evenness |
| --- | --- | --- | --- | --- |
| A | 1.42^a^ | 0.35^a^ | 1.41^a^ | 0.67^a^ |
| B | 1.08^b^ | 0.61^b^ | 0.84^b^ | 0.42^b^ |
| C | 1.18^b^ | 0.59^b^ | 0.88^b^ | 0.42^b^ |
